# Do hearing loss interventions prevent dementia?

**DOI:** 10.1007/s00391-023-02178-z

**Published:** 2023-05-04

**Authors:** Piers Dawes, Christiane Völter

**Affiliations:** 1 University of Queensland Centre for Hearing Research (CHEAR), School of Health and Rehabilitation Sciences, The University of Queensland, 4072 Brisbane, QLD Australia; 2 Manchester Centre for Audiology and Deafness (ManCAD), The University of Manchester, Manchester, UK; 3 Department of Otorhinolaryngology, Head and Neck Surgery, Comprehensive Hearing Centre at Katholisches Klinikum, Ruhr-University, Bochum, Germany

**Keywords:** Review, Cognitive impairment, Hearing aids, Cochlear implants, Risk factors, Übersichtsarbeit, Kognitive Beeinträchtigung, Hörgeräte, Cochlea-Implantate, Risikofaktoren

## Abstract

Hearing loss has been identified as a potentially modifiable risk for dementia. This discussion paper reviews studies examining the impact of hearing loss interventions on cognitive decline and incident cognitive impairment, identified the challenges for research on the cognitive impacts of hearing interventions, and the likely benefits of hearing interventions for healthy aging and mental well-being.

**Supplementary Information:**

The online version of this article (10.1007/s00391-023-02178-z) contains supplementary material, which is available to authorized users.

## Abstract (de)

Eine Hörminderung gilt als einer der möglichen Risikofaktoren für das Auftreten einer Demenz. Die vorliegende Arbeit soll einen Überblick über die aktuellen Studien zu den Auswirkungen hörrehabilitativer Maßnahmen auf den kognitiven Abbau im Alter geben und kritisch die Herausforderungen und Bias in diesem Kontext sowie den Benefit einer adäquaten Hörversorgung im Hinblick auf ein gesundes Altern und das geistige Wohlbefinden darlegen.

Due to aging populations and increasing numbers of people living with dementia, identifying treatments and strategies to prevent or delay the onset of dementia is a global priority. One report concluded that delaying the onset of dementia by only 2 years would reduce the prevalence of dementia by 20% [1]. Healthy lifestyle interventions offer an opportunity to prevent or delay dementia [2]. The 2020 Lancet Commission report on dementia prevention and care [3] summarized various potentially modifiable lifestyle factors and long-term medical conditions linked to risk of dementia. The report concluded that if one could eliminate all these health and lifestyle risks, one could theoretically prevent up to 40% of cases of dementia. Hearing loss was one of the largest risks that the report identified, with 8% of cases of dementia attributable to hearing loss in mid-life. This statistic suggests that if hearing loss could be eliminated or entirely mitigated, this would lead to an 8% reduction in the number of cases of dementia.

This frequently quoted 8% statistic deserves some explanation. First, this statistic does not describe the strength of the risk of dementia at an individual level as sometimes stated [4], but the proportion of cases of dementia associated with hearing loss for the general population, the percent attributable fraction. The percent attributable fraction for each risk factor is a function of the strength of the risk factors’ association with incident dementia by the prevalence of each factor (i.e., the level of exposure to each risk within the population). The percent attributable fraction for hearing loss is relatively high partly because hearing loss is so prevalent in the population. Other factors (e.g., depression) were associated with a similar size of risk of dementia as hearing loss was at the individual level [3].

Second, the calculation of the attributable fraction of dementia cases for each factor in the Lancet report was based on the levels of exposure for those factors in the United Kingdom (UK) and similar high-income countries. Levels of exposure to each risk factor and the relevant risk factors are different across countries, and so the attributable fraction of dementia cases is also different. For example, the level of tobacco smoking in China is around 2.5 times higher than in the UK. Smoking is therefore probably a much more important contributor to dementia risk in China than in the UK. Different risk factors may be important in low-income and middle-income countries, such as maternal and early life malnutrition. Levels of hearing loss may also be different across countries [5]. It is incorrect to assert that hearing loss is the leading potentially modifiable risk for dementia because that may only be valid for high-income countries like the UK. As the numbers of people living with dementia and the projected increases in numbers of people with dementia is highest for low-income and middle-income countries [6], it is problematic to focus on risk factors relevant for high-income countries.

Finally, the conclusion that 8% of dementia cases are associated with hearing loss was based on a meta-analysis of 3 studies [7–9] that linked baseline levels of hearing loss to risk of subsequent dementia. Because these were observational studies, they could not establish a causal relationship. There are three scenarios that may explain an association between hearing loss and dementia risk (Fig. 1). First, hearing loss could plausibly impact on cognition, either directly via alternations in auditory input impacting on brain structures that support cognition, or indirectly via increased social isolation, depression, reduced self-efficacy, reduced physical activity, or reduced participation in cognitively stimulating activities [10–12]. If there is a direct or indirect causal impact of hearing loss on cognition, one might expect that interventions to address hearing loss might have a beneficial effect on cognition [13].

**Fig. 1:**
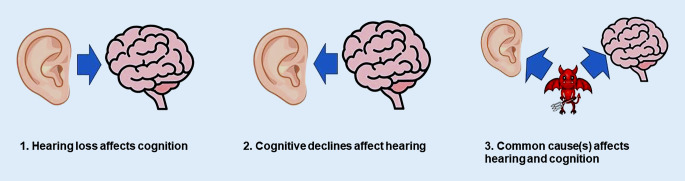
Three scenarios that may explain an association between hearing loss and dementia risk

But an alternative explanation for hearing loss being associated with cognitive decline and risk of dementia is that cognitive declines impact hearing [14]. Listening is cognitively demanding [15]. Someone might have hearing problems due to cognitive decline, rather than hearing affecting cognition.

Lastly, hearing loss and dementia may be associated due to a common cause. Hearing loss could be a marker of neurological frailty due to disease processes that impact on both hearing and cognition [16]. All the lifestyle and health risk factors for dementia that the Lancet Commission identified are also risk factors for hearing loss (e.g. physical inactivity, smoking, poor diet, poor air quality, excessive alcohol consumption, low educational level, high blood pressure, obesity, high cholesterol, and diabetes). Observational studies examining relationships between hearing loss and dementia try to measure and control for these shared factors, but some factors may not have been measured, or incompletely accounted for.

Lastly, hearing tests may be confounded with cognitive factors. Cognitive factors may impact performance of hearing tests [17] and cognitive difficulties increase the likelihood of reporting hearing difficulties [18]. Conversely, hearing affects the performance of cognitive assessments [19] and tests for cognitive impairment [20]. The degree of impact depends on the severity of hearing impairment and the task demands of the cognitive test. Confounding of hearing and cognition may overestimate the association between hearing, cognitive performance, and risk of dementia.

There is evidence for all the above potential relationships between hearing and cognitive outcomes. Recent studies [21, 22] that simultaneously modelled alternative explanations for the association between hearing loss and cognition suggest that there is both a common cause and additional impacts of hearing loss on cognition. To summarize, the association of hearing loss with the risk of dementia is smaller at an individual level than perhaps implied by the attributable fraction statistics for the general population in the Lancet report. Hearing loss may impact on cognition, but at least some of the association between hearing loss and risk of dementia is probably due to common causes and confounding of cognition and hearing measures; however, there are plausible hypotheses for how hearing impairment may adversely impact cognition and risk of dementia.

A key question then, is whether treating the hearing impairment (e.g., with hearing aids or cochlear implants) reduces the risk of dementia. Several prospective hearing aid and cochlear implant trials measured cognitive outcomes. Some reported improvement, some no change and some reported worse cognitive outcomes following hearing intervention [23]. Most studies are limited by their short duration, typically a few weeks up to 18 months. As cognitive decline is gradual [24], studies need to be of much longer duration to observe the effect of hearing intervention on cognitive decline. The first author (PD) previously reviewed 7 studies of hearing interventions with cognitive outcomes assessed over longer than 3 years [25]. Three years was chosen as the minimum follow-up because it is conceivable that cognitive decline may be observable during time scales of at least 3 years [26]. In the current review, we provide an update and overview of 18 studies of hearing interventions for adults with normal cognition that evaluated cognitive outcomes (including cognitive change and incident cognitive impairment) over durations longer than 3 years that have been published up to December 2022 (that we are aware of; Supplementary Material Tables 1 and 2). We chose to include studies that examined both incident cognitive impairment and change in cognitive performance, because the latter (i.e., cognitive decline) is a risk factor for dementia, and to include studies that examined benefits of hearing interventions on long-term cognitive change. Three studies reported cochlear implants and 15 reported hearing aid interventions. All of the studies were of low to moderate level of evidence [27]; studies were mostly single group interventions or observational designs.

## Hearing aids

Four studies reported lower incident cognitive impairment among hearing aid users compared to non-users (mild cognitive impairment [28] or dementia [29–31]). Another five studies reported no differences in incident cognitive impairment (dementia [32, 33], cognitive impairment identified by a screening test [34, 35] or subjective cognitive impairment [36]). Four studies reported reduced cognitive decline among hearing aid users [37–40] while another three studies reported no difference in cognitive decline for hearing aid users versus non-users [34, 41, 42]. Differences between studies may be related to variation in sample characteristics, measures of hearing and cognition, length of follow-up, selection of statistical models and range and adequacy of control for confounding factors.

All these studies involved statistical comparison of cognitive outcomes for hearing aid users versus non-users in longitudinal data sets. The advantages of observational studies with existing data sets are that they provide immediate answers to questions about the benefits of hearing interventions, rather than having to wait for the results of a prospective trial. They also have ecological validity in that they utilize real world data rather than from a trial of an intervention that may not be widely implementable in practice. One limitation of some studies is that they rely on self-reporting [28, 35, 36, 40] or health records [29–31] to index hearing loss rather than gold standard audiometric assessment used by other studies [32, 34, 37–39, 41, 42]. Self-reported hearing underestimates actual levels of impairment; however, the main difficulty with observational studies is the potential for confounding of hearing aid use with other factors that may impact cognitive outcomes. Only around 10–20% of people with hearing loss use hearing aids [43]. Hearing aid users tend to be more affluent, better educated and more likely to be members of majority ethnic groups than non-hearing aid users [43]. The observational studies in this review minimized the impact of demographic and health confounds by statistical adjustment; however, it is possible that the better outcomes for hearing aid users observed in some studies are due to unmeasured confounds, or to imperfect control for measured confounds (i.e., residual confounding). For example, although Mahmoudi et al. statistically controlled for large differences in sex (lower use among women) and ethnicity (lower use among Black and Latino people) between hearing aid users and non-users [31], they lacked data on educational level and socioeconomic status, which are key correlates of health outcomes and a potential confound.

A recent meta-analysis synthesized the results of 8 hearing aid studies with between 2 and 25 years of follow-up and estimated a lower hazard of incident cognitive impairment (based on clinical diagnosis of dementia or measurement of cognitive function, e.g., with the MMSE) among hearing aid users versus non-users (HR 0.81, 95% CI 0.76–0.87; a weak association [44]). It is promising that the overall pattern of findings suggests a benefit of hearing aids but the limitations described above also apply to this meta-analysis, i.e., there is potential for residual confounding. Due to its large sample size, the analysis by Mahmoudi et al. [31] was the most influential contributor to the meta-analysis although it lacked control for key confounds. Creative study designs may mitigate the impact of residual confounding. For example, Maharani et al. [37] compared rates of decline in memory before and after hearing aid use in the same individuals. Encouragingly, Maharani et al. reported a slower rate of memory decline following hearing aid use.

## Cochlear implants

Outcomes vary but on average cochlear implants restore functional hearing to a person with profound hearing loss [45]. In contrast, hearing aids provide an incremental increase in audibility of sounds that were partially audible prior to using the hearing aid for people with mild to moderate hearing loss. One might therefore hypothesize a larger impact of cochlear implants on cognitive outcomes than hearing aids. Results from three cochlear implant studies are encouraging, but methodological shortcomings (including a lack of satisfactory control group comparison and high attrition rates) preclude conclusions about the cognitive impacts of cochlear implantation.

Cosetti et al. [46] reported improvements on a cognitive test battery among 7 women an average of 3.7 years after cochlear implantation. Cosetti et al. reported that 20 other participants who were assessed at baseline had dropped out (70% attrition rate). The very high attrition rate raises potential for bias. Mosnier et al. [47] tested 70 cochlear implant recipients up to 7 years post-implant in a multicenter study. Based on a previous report with this cohort [48] 24 participants were lost to follow-up (26% attrition rate). Mosnier et al. reported significant declines in performance in 5 out of 7 cognitive tests, with no change in the remaining two tests. Mosnier et al. also reported that the incidence of mild cognitive impairment (MCI) and dementia was lower than might have been expected based on population data for incidence of cognitive impairment; however, rates of cognitive impairment were not tested statistically versus general population estimates. It was unclear why there might be lower incidence of cognitive impairment among cochlear implant users than among the general population. Furthermore, diagnosis of cognitive impairment included performance on study cognitive tests which were repeatedly administered and may be subject to practice effects. Both the Mosnier et al. and Cosetti et al. studies were single group designs, making it difficult to disentangle practice effects from genuine improvements in cognition. Practice effects on cognitive tests are known to be substantial and long-lasting [49].

Völter et al. [50] attempted a controlled comparison of cognitive changes over an average of 5 years between 50 cochlear implant recipients and an age-matched and educational level-matched cohort of adults (*n* = 1000) from the Survey of Health Ageing and Retirement in Europe (SHARE). Völter et al. reported improvements in a delayed recall task among cochlear implant recipients, with a slight decline in performance on a delayed recall task among SHARE participants. Cochlear implant recipients improved on a working memory task, while there was no change in a working memory task among SHARE participants. Cognitive changes among cochlear implant recipients were not directly statistically compared with changes in the SHARE cohort. Unfortunately, different tests were used in the SHARE cohort to the cochlear implant recipients. Although cognitive tests may purport to assess the same cognitive domain, they may have different sensitivity to cognitive changes over time. Additionally, 21 cochlear implant recipients had dropped out since baseline (30% attrition rate), which may positively bias outcomes. Völter et al. reported that there was no correlation between improvement in speech perception and improvements in cognitive performance, and most improvements occurred within the first year after cochlear implantation. Völter et al. suggested that cochlear implantation and the subsequent rehabilitation program might have an indirect effect that positively impacted cognition (e.g,. a more active lifestyle or an increase in stimulating social interactions).

## Discussion

Literature on the benefits of cochlear implantation on cognitive decline and incident cognitive impairment is ambiguous. The hearing aid literature is approximately equally divided between studies that reported a benefit and those that did not. The possibility of preventing or delaying dementia by treating hearing impairment is appealing, but there are challenges in understanding the benefits of hearing interventions.

Several studies examined hearing intervention in relation to all cause incident dementia [29–32]. Dementia is a symptomatic description for cognitive impairment that may result from a range of pathologies. One might hypothesize that hearing loss may have different relationships with different types of dementia, and that hearing interventions could have more or less impact according to dementia type [10, 12, 51]. A limitation of existing literature might be availability of data concerning dementia subtypes and/or sufficient sample size to support subtype analysis. Several studies utilized health insurance claims data for large national data sets [29–31], which may offer sufficient sample size. Unfortunately, no study reported analyses according to dementia subtypes.

Other studies reported performance on a variety of cognitive outcome measures indexing a range of cognitive domains including attention, processing speed, executive functioning, short-term memory, working memory and long-term memory. The choice of cognitive outcome measures in each study appears to be predominantly based on what data were available. Some researchers hypothesize different effects of hearing loss on specific cognitive domains [52–54]. Ronnberg et al. [52], for example, hypothesized effects of hearing loss on episodic long-term memory. A meta-analysis reported that correlation with hearing impairment was similar across cognitive domains [23]. Future studies could consider a rationale for choice of cognitive measures based on hypothesized causation and/or clinical or functional relevance. Some studies [40] used screening tests for cognitive impairment to index cognitive performance (e.g. ,the mini mental status examination [MMSE]). Tests like the MMSE are designed to detect gross cognitive impairment and are relatively insensitive to subtle cognitive changes. Several studies [32, 34, 35, 38, 40, 41, 46, 47, 55] included spoken tests of cognition, or relied on the record of a clinical diagnosis of cognitive impairment [28–31, 39, 42]. Hearing impairment impacts performance on tests for cognitive impairment [20, 51] and other cognitive performance tests [19]. Hearing checks are not typically carried out during dementia diagnosis [56], and hearing loss may increase the likelihood of dementia diagnosis [57]. Confounds of hearing loss with spoken cognitive tests and clinical diagnoses of cognitive impairment may lead to overestimation of the impact of hearing interventions. That is because the apparent benefit of hearing interventions may be due to restoration of audibility of spoken cognitive tests, rather than an impact on cognition. Using a common assessment (such as the NIH toolbox [58]) and visually presented tests (such as the hearing-impaired version of the Montreal cognitive assessment [59]) would facilitate comparison across studies and reliable assessment of cognition among people with hearing loss.

Specific hypotheses about the relationship between hearing loss and cognitive outcomes and the mechanisms of effect of hearing interventions on cognition could be tested in longitudinal observational studies, for example using structural equation methodology [60]. Unfortunately, although studies contained data concerning a potential common cause, psychosocial or behavioral mediators (e.g., long-term health conditions, social isolation, depression, exercise, smoking, alcohol consumption), these factors were treated as confounds rather than potential causal mediators. Causal modelling may inform debate about the causal relationship between hearing loss and dementia and inform prospective intervention studies.

The manuscripts described in the supplemental tables typically call for prospective randomized controlled trials to provide a rigorous analysis of the impact of hearing interventions on cognitive outcomes. Such trials are under way [61–63], but they also have limitations. Ideally, trials would address the question of whether hearing interventions prevented or delayed dementia but because of the unfeasibly large size of a study powered to detect differences in incidence of dementia in cognitively normal populations, trials instead focus on change in cognitive performance. There are also challenges with selective drop-out, practice effects and adherence with hearing interventions. Furthermore, because the benefits of hearing interventions for people with hearing loss are well-established [45, 64], there is an ethical issue with providing hearing treatment to an intervention group while withholding treatment from a control group. Studies have dealt with this issue by being relatively short in duration [62] (which may limit the potential to observe an impact on cognitive decline) or comparing hearing intervention to an alternative healthy living intervention [38]. Healthy lifestyle intervention is one of the few things that is effective in improving cognitive outcomes and preventing dementia [2]. Interpretation of a trial comparing a hearing intervention to a healthy lifestyle intervention may therefore be problematic. Randomized controlled trials are desirable, but they might not deliver the definitive results expected. Public health decisions about the utility of hearing interventions for dementia prevention may therefore be informed by a combination of experimental and observational studies.

We focused on device-based hearing interventions (cochlear implants and hearing aids), as they are the commonest interventions for hearing loss. There are also interventions that address the communication difficulties and psychosocial impacts of hearing loss with counselling and problem solving (e.g., active communication education; ACE [65]). Although effective [65], these types of interventions tend to be neglected in favor of device-based interventions. We are not aware of any evaluations of the ACE or similar interventions on incident dementia or other cognitive outcomes. The ACE and similar interventions may be effective in relation to dementia prevention, especially if the causal pathway between hearing loss and dementia is via psychosocial factors (e.g., depression, social isolation).

The jury is out on the benefits of hearing interventions in reducing the risk of dementia. The current WHO guidelines for risk reduction of cognitive decline and dementia say that there is insufficient evidence to recommend use of hearing aids to reduce the risk of cognitive decline and/or dementia [66]. The practice of promoting hearing interventions as reducing the risk of dementia [4] is problematic because the benefit of hearing interventions has not been established in this respect. With good intentions, hearing aid manufacturers and clinicians may be keen to make a link between hearing loss and dementia risk to motivate people with hearing loss to seek help for hearing problems. People may (incorrectly) see hearing loss as an inconsequential problem and an inevitable aspect of older age that one cannot do much about [67]. But linking hearing loss to dementia may have unintended consequences in making people less likely to engage with hearing interventions [68]. That is because linking hearing loss to dementia may reinforce stigma and resultant sense of personal threat, denial and disengagement with hearing care that people may experience in relation to hearing loss diagnosis [69]. Clinicians should be appropriately cautious about overstating the risks of hearing loss for dementia and the benefits of hearing interventions in preventing dementia; however, there are clear benefits of hearing aids and cochlear implants in improving communication and facilitating an active, socially engaged, healthy lifestyle [45, 64].

**Fig. 2:**
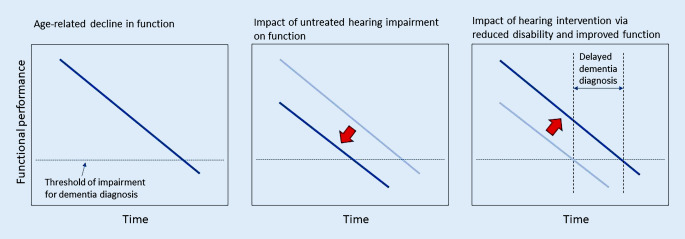
Impact of untreated hearing impairment and benefits for hearing intervention on function and progression to dementia. *Dark* and *light blue lines* indicate trajectories of functional decline with age-related changes in cognition

In our opinion, the key benefit of hearing interventions in preventing dementia is probably via the interaction with hearing and cognition on functioning in daily life. Dementia is diagnosed based on cognitive impairment severe enough to impact on independent functioning in daily life [70] (the left box in Fig. 2). Hearing loss also impacts on functional ability and exacerbates the impacts of cognitive impairment [71] (the middle box in Fig. 2). Hearing interventions reduce disability and improve function in daily life [45, 64] (the right box in Fig. 2). Hearing interventions therefore probably do help prevent dementia diagnosis via decreasing or reversing disability. Hearing interventions are therefore likely effective in delaying the point at which cognitive difficulties become dementia, preserving independence, social engagement, and quality of life. Such a benefit would be independent of the type of dementia pathology. This possibility could be tested via assessing functional consequences of hearing interventions, interactions with cognitive status and how this relates to subsequent dementia diagnosis.

## Conclusion

Clinicians who work with older people should discuss hearing interventions in the context of healthy living, and how communication is vital in facilitating an active independent and vibrant lifestyle. Orienting the discussion around hearing interventions as healthy lifestyle choices (rather than stigmatizing medically focused interventions in the context of dementia risk) may help put an appropriately positive spin on hearing care and encourage engagement and uptake of hearing interventions. An appealing proposition for people with hearing difficulties is that the benefits of hearing interventions extend beyond improved communication.

## Supplementary Information

Summary tables of hearing aid and cochlear implant intervention studies for adults with normal cognition with cognitive outcomes assessed over greater than 3 years

## Declarations

For this article no studies with human participants or animals were performed by any of the authors. All studies mentioned were in accordance with the ethical standards indicated in each case.
