# Supplementary material for: Do hearing loss interventions prevent dementia?
Source: Z Gerontol Geriatr. 2023 May 4;56(4):261–8. doi: 10.1007/s00391-023-02178-z (PMC10289956; doi:10.1007/s00391-023-02178-z)
Supplement: Supplementary file 1 — Summary tables of hearing aid and cochlear implant intervention studies for adults with normal cognition with cognitive outcomes assessed over greater than 3 years [file 391_2023_2178_MOESM1_ESM.docx]

**Supplementary Material**

**Table 1.** Hearing aid intervention studies for adults with normal cognition with cognitive outcomes assessed over greater than 3 years

| **Study** | **Study design*** | **Participants** | **Outcome measures** | **Results** | **Covariates controlled for** |
| --- | --- | --- | --- | --- | --- |
| **Hearing aids** | | | | |  |
| Byun et al. (2022) | Level 3 (Non-randomized controlled cohort) | Adults aged 30-80 years registered with a hearing disability (better year >40 dB HL) according to Korean National Health Insurance System data; 8780 who obtained hearing aids and a non-hearing aid cohort matched for age, sex, income, urban/rural setting, number of comorbid health conditions, year of disability registration and severity of hearing loss. | Incident all-cause dementia based on Korean National Health Insurance System record; mean follow-up time 10.5 and 9.7 years for the hearing aid and non-hearing aid cohorts, respectively. | Hearing aid users were less likely to develop dementia than non-users (HR 0.75, 95% CI 0.70-0.81) | Age, sex, income, urban/rural setting, number of comorbid health conditions, year of disability registration and severity of hearing loss. |
| Sugiura et al.(2022). | Level 3 (Non-randomized controlled cohort) | Adults aged over 60 years with audiometric hearing loss (better ear threshold >25 dB HL) (n=1193) including 71 hearing aid users. (Japanese National Institute for Longevity Sciences Longitudinal Study of Aging) | Cumulative cognitive impairment, based on Mini Mental Status Examination score <27 or reported doctor diagnosis of dementia. | No association between hearing aid use and cognitive impairment (OR 0.90, 95% CI 0.63-1.28).  No association whether or not occasional HA users were included. | Age, sex, depression, smoking, alcohol intake, education, obesity, physical activity, hypertension, hypercholesterolemia, diabetes, heart disease, stroke, ear disease and occupational noise exposure |
| Bucholc, Bauermeister et al. (2021) | Level 3 (Non-randomized controlled cohort) | Adults over 40; 450 with self-reported hearing impairment including 137 hearing aid users; 3,908 with self-reported normal hearing from the United States National Alzheimer’s Coordinating Center database. | Incident mild cognitive impairment based on clinician diagnosis; mean follow-up time 4 years (standard deviation 2.8; range 2 to 12). | Among those with hearing impairment, hearing aid users less likely to develop MCI than non-users (HR 0.47, 95% CI 0.29-0.74).  No difference in risk of MCI for hearing aid users versus those with normal hearing. | Age, sex, education, smoking, hypertension, diabetes, hypercholesterolemia, alcohol dependence, stoke, heart attack/cardiac arrest, BMI, depression, and selection bias due to drop-out. |
| Sugiura et al. (2021). | Level 3 (Non-randomized controlled cohort) | Adults aged over 60 years with moderate hearing loss (40-69 dB HL in the better ear; n = 407), including 128 hearing aid users (Japanese National Institute for Longevity Sciences Longitudinal Study of Aging). | Cognitive function indexed by the Wechsler Adult Intelligence Scale-Revised Short Forms (Information; Similarities; Picture completion; Digit Symbol Substitution) at baseline and up to 15 years follow up (mean follow-up 4.5 SD 3.9 years) | In general linear mixed models, there was a significant time*hearing aid use interaction for the Information subtest (β=0.100; i.e. less decline among hearing aid users).  There was no time*hearing aid use interaction for Similarities, Picture completion or Digit Symbol Substitution. | Age, sex, education, marital status, occupation, income, depression, smoking, obesity, hypertension, diabetes, hypercholesterolemia, heart disease and stroke. |
| Tai et al. (2021). | Level 3 (Non-randomized controlled cohort) | Adults aged over 60 years with self-reported hearing loss (n=775, including 83 hearing aid users) and those with normal hearing (n=709) matched for age, comorbid health conditions, socioeconomic status, social connections, use of assistive devices, instrumental activities of daily living, depression, mobility status and cognitive performance (SPMSQ) (Taiwan Longitudinal Study on Aging) | Incident cognitive impairment based on Short Portable Mental Status Questionnaire (SPMSQ) with a mean follow-up of 8.9 (SD 3.9) years. | People with hearing impairment were more likely to experience cognitive impairment (HR 1.16, 95%CI 1.03-1.32). Among those with hearing impairment, there was no significant difference in incidence of cognitive impairment between hearing aid users and non-users. | Age, comorbid health conditions, socioeconomic status, social connections, use of assistive devices, instrumental activities of daily living, depression, mobility status and cognitive performance (SPMSQ). |
| Curhan et al. (2019). | Level 3 (Non-randomized controlled cohort) | Men aged ≥62 years (n=10,107) who reported hearing status in 2006 (according to ‘no hearing difficulty’ (n=5403), ‘mild’ (n=2836), ‘moderate’ (n=1049), ‘severe; no hearing aid’ (n=196) and ‘severe; use a hearing aid’ categories (n=623)) and reported no subjective cognitive concerns in 2008 (Health Professionals Follow-up Study) | Incident subjective cognitive decline (defined as at least one new report of concern on a 6-item subjective cognition function questionnaire) 2008 baseline up to 2016 (8-year follow-up). | Compared with no hearing difficulty, relative risk (RR) of incident subjective cognitive decline was higher in those with mild, moderate and severe hearing loss (no hearing aids) (RR’s and 95% CI’s 1.30, 1.18-1.42; 1.42 (1.26-1.61); 1.54 (1.22-1.96), respectively. Relative risk of subjective cognitive impairment was also higher among those with severe hearing loss who used hearing aids (RR 1.37, 95% CI 1.18-1.60), not statistically significantly different from non-hearing aid users. | Age, race, occupation, BMI, waist circumference, smoking, physical activity, hypertension, diabetes, hypercholesterolemia, depression, aspirin use, ibuprofen use, acetaminophen use and dietary intake. |
| Mahmoudi, et al. (2019). | Level 3 (Non-randomized controlled cohort) | Adults over 66 (n=114862, including n=14 109 hearing aid users) identified with hearing loss based on US health insurance claim data | Incident all-cause dementia recorded from health insurance claim data within 3 years of hearing loss diagnosis. | Hearing aid users were less likely to be diagnosed with dementia than non-users (HR 0.82, 95% CI 0.76 to 0.89). | Age, sex, ethnicity, census division, cardiovascular condition, hypertension, hypercholesterolemia, obesity and diabetes. |
| Maharani et al. (2018). | Level 3 (Non-randomized controlled cohort) | Adults over 50 who began using a hearing aid between 1996 and 2014 | Episodic memory scores assessed every 2 years (between 6 to 9 years follow-up) | Reduction in rate of decline in memory following commencement of hearing aid use (β=-0.02 vs -0.10) | Age, sex, education, marital status, smoking, alcohol consumption, physical activity, depression score and health comorbidities |
| Davies et al. (2017) | Level 3 (Non-randomized controlled cohort) | Adults aged over 50 (n=8,651) including those with ‘moderate’ or ‘poor’ self-reported hearing (n=4,461) and hearing aid users (n=532) (English Longitudinal Study of Aging) | Participant-reported doctor diagnosis of incident dementia between 2004/5 and 2014/15. | No differences between hearing aid users and non-users in incidence of dementia. | Age, sex, wealth, education, hypertension, diabetes, and stroke history |
| Fritze et al. (2016) | Level 3 (Non-randomized controlled cohort) | Adults over 65 (n=154,783), including with those with bilateral hearing loss (n=91,421) and those who received treatment by an ENT doctor (n=5231), identified based on German health insurance claim data | Incident all-cause dementia recorded from health insurance claim data between 2006 and 2010. | Bilateral hearing loss was associated with increased risk of dementia (HR 1.16; 95% CI not reported). Having received treatment by an ENT doctor was associated with a reduced risk of dementia (HR 0.74, 95% CI not reported). | Age, gender, tinnitus and ‘comorbidities’ |
| Amieva et al. 2015 | Level 3 (Non-randomized controlled cohort) | 150 hearing aid users; 1,126 adults self-reported hearing loss (HL); 2,394 ‘adults with self-reported normal hearing (NH), all aged >65 years at baseline | Mini Mental State Examination (MMSE), approximately every three years for 25 years | Slower rate of decline for hearing aid users verses NH (difference in rate of decline: β=0.05) | Age, sex, educational level, depression, living situation, social network, comorbidities, medication, dementia |
| Deal et al. 2015 | Level 3 (Non-randomized controlled cohort) | Adults with moderate/severe hearing impairment (better ear >40dB HL); 42 non-hearing aid users and 43 hearing aid users | Three cognitive tests in 1990-92, 1996-98 and 2013; Memory (Delayed Word Recall), language (Word Fluency), processing speed/attention (Digit Symbol Substitution Test) | Slower decline for hearing aid users versus non-users (β=-1.45 versus -0.97) on cognitive composite score. | Age, age^2^, sex, education, smoking status, diabetes, hypertension, and Wide Range Achievement Test score |
| Dawes et al. 2015 | Level 3 (Non-randomized controlled cohort) | Adults with hearing impairment (better ear >40dB HL); 597 non-hearing aid users and 69 hearing aid users, average age 68 years at baseline. | MMSE at 5 and 11 years after baseline; Trial Making, Auditory Verbal Learning Test, Digit Symbol Substitution Test, Verbal Fluency at 11 years post baseline  Incident cognitive impairment (reported diagnosis or MMSE<24) | No differences between hearing aid users and non-users in cognitive tests or incidence of cognitive impairment at any time point. | Age, sex and hearing level |
| Lin et al. 2013 | Level 3 (Non-randomized controlled cohort) | Adults with hearing impairment (better ear 40-70dB HL; based on audiometric evaluation at year 5 of a 6 year-follow-up); 218 non-hearing aid users and 182 hearing aid users, average at 78 at baseline | 3MS (global function) and Digit Symbol Substitution (processing speed/attention) 6 years post baseline  Incident cognitive impairment (3MS score<80 or decline in 3MS >5 points from baseline) | No differences between hearing aid users and non-users in rate of decline or incidence of cognitive impairment. | Age, sex, ethnicity, education, study site, smoking, hypertension, diabetes, and stroke history |
| Valentijn et al. 2005 | Level 3 (Non-randomized controlled cohort) | 391 adults with mean hearing level 16 dB HL (range 0 to 58) at baseline, including 7 who obtained a hearing aid between baseline and follow-up | MMSE, Visual Verbal Learning Test, Stroop Color Word Test, Concept Shift Test, Verbal Fluency, Letter-Digit Substitution at baseline and 6 year-follow-up | No interaction with hearing aid use and cognitive change at 6 year-follow-up | Age, sex, and education |

*According to the *Oxford 2011 Levels of Evidence* (OCEBM Levels of Evidence Working Group, 2011, which are: Level 1, fully powered randomised controlled trials or meta-analysis; Level 2, controlled trials without randomisation; Level 3, retrospective cohort or case-control studies; Level 4, case series or uncontrolled single group study; and Level 5, expert opinion or case report).

**Table 2.** Cochlear implant intervention studies for adults with normal cognition with cognitive outcomes assessed over greater than 3 years

| **Cochlear implants** | | | | |
| --- | --- | --- | --- | --- |
| **Study** | **Study design*** | **Participants** | **Outcome measures** | **Results** |
| Völter et al. (2022) | Level 3 (Non-randomized controlled cohort) | Adult cochlear implant recipients (n=50) aged ≥50 years (average age 64.4); Comparison cohort (n=1,000) of adults matched for age and educational level from the Survey of Health, Ageing and Retirement in Europe (SHARE) | Cochlear implant: 30-minute delayed recall, working memory (2-back letter sequence task and operation span), visual attention, inhibition (Erikson flanker task), verbal fluency and trial making test prior to implantation, pre-operative baseline and 4.5 (standard deviation 0.5) year follow-up post implantation.  SHARE comparison cohort: 10-minute delayed recall, Serial 7s task (working memory) at 2015 baseline and 2020 follow-up. | Improvement in visual attention, inhibition, delayed recall, operation span (medium effect sizes; *d’*s 0.5-0.58), and verbal fluency (small effect size *d* = 0.43).  No change in 2-back task or trial making tests.  Cochlear implant recipients improved on the 30-minute delayed recall task (β=0.169), while there was a decline in performance on the 10-minute delayed recall task in the SHARE cohort (β=-0.076).  Cochlear implant recipients improved on the operation span task (β=0.167), while there was no significant change performance in the SHARE cohort. |
| Mosnier et al. (2018) | Level 4 (Uncontrolled single group study) | Adult cochlear implant recipients (n=70; including n=30 with MCI) aged >65 (average age 72 at baseline) | Cognitive test battery (Mini Mental State Examination [MMSE], 5-word test, clock drawing, d2 Test of Attention, Trail Making, Fluency) at 1 year and 7 years post implantation  Dementia status, determined by a physician based on  i) significant impairment in social and activities of daily living and ii) worse performance on 2 or more tests from the cognitive test battery 7 years vs 1 year  MCI, greater degree of cognitive decline ‘than expected for age’ on the cognitive test battery without impairment in daily functioning | *Cognitive test battery*  Worse performance (MMSE, Clock Draw, d2 Test of Attention, TMT, Fluency letters); no difference (5-word test, Fluency categories) between 1 and 7 years post implantation  *Dementia/MCI*  Of 31 participants with MCI at baseline, 2 developed dementia.  Of 38 participants with normal cognition 12 developed MCI. |
| Cosetti et al. 2016 | Level 4 (uncontrolled single group study) | Adult cochlear implant recipients (n= 7) aged ≥67 years (mean age 73.6 at baseline) | Cognitive test battery (Test of Premorbid Functioning (TOPF), the Wechsler Abbreviated Scale of Intelligence (WASI), Trail Making Test (TMT), Controlled Oral Word Association Tests, Boston Naming Test (BNT), Repeatable Battery for the Assessment of Neuropsycho­logical Functioning (RBANS)) preoperatively and 2 to 4.1 years after implantation (mean follow-up 3.7 years). | Significant improvement reported for TOPF, WASI-verbal IQ and full-scale IQ, Vocabulary, Similarities, Matrix Reasoning, BNT, Picture Naming and List learning; no significant change on two remaining subtests (List recall and WASI performance IQ), according to mean ‘percentage improvement’ and *p* values from a logistic regression model |
